# Quantitative T_1_ and Effective Proton Density (PD*) mapping in children and adults at 7T from an MP2RAGE sequence optimised for uniform T_1_-weighted (UNI) and FLuid And White matter Suppression (FLAWS) contrasts

**DOI:** 10.1162/IMAG.a.966

**Published:** 2025-11-13

**Authors:** Ayşe Sıla Dokumacı, Katy Vecchiato, Raphael Tomi-Tricot, Michael Eyre, Philippa Bridgen, Pierluigi Di Cio, Chiara Casella, Tobias C. Wood, Jan Sedlacik, Tom Wilkinson, Sharon L. Giles, Joseph V. Hajnal, Jonathan O’Muircheartaigh, Shaihan J. Malik, David W. Carmichael

**Affiliations:** Imaging Physics and Engineering Research Department, School of Biomedical Engineering and Imaging Sciences, King’s College London, London, United Kingdom; London Collaborative Ultra high field System (LoCUS), London, United Kingdom; Department of Forensic and Neurodevelopmental Sciences, Institute of Psychiatry, Psychology and Neuroscience, King’s College London, London, United Kingdom; Early Life Imaging Research Department, School of Biomedical Engineering and Imaging Sciences, King’s College London, London, United Kingdom; MR Research Collaborations, Siemens Healthcare Limited, Frimley, United Kingdom; Children’s Neurosciences, Evelina London Children’s Hospital at Guy’s and St Thomas’ NHS Foundation Trust, London, United Kingdom; Guy’s and St Thomas’ NHS Foundation Trust, London, United Kingdom; Department of Neuroimaging, Institute of Psychiatry, Psychology and Neuroscience, King’s College London, London, United Kingdom; Robert Steiner MR Unit, Medical Research Council Laboratory of Medical Sciences, Hammersmith Hospital Campus, London, United Kingdom; Mansfield Centre for Innovation, Imaging Sciences, Institute of Clinical Sciences, Imperial College London, Hammersmith Hospital Campus, London, United KIngdom; MRC Centre for Neurodevelopmental Disorders, King’s College London, London, United Kingdom

**Keywords:** MP2RAGE, quantitative MRI, 7T, ultra-high field, T_1_ mapping, PD mapping

## Abstract

Quantitative T_1_ and PD mapping is important for non-invasive tissue characterisation. Here, we aimed to calculate effective proton density (PD*) and quantitative T_1_ relaxation maps at 7T using the MP2RAGE sequence and an analytical formula. This was applied to a short repetition time (TR_MP2RAGE_) protocol developed to produce both Fluid And White Matter Suppression (FLAWS) and T_1_-weighted uniform image (UNI) images. T_1_ and PD* were estimated from analytical MP2RAGE signal equations for partial Fourier acquisitions. The sensitivity with respect to the TR_MP2RAGE_ and B_1_^+^ effects on both excitation flip angles and inversion efficiency was evaluated. T_1_ and PD* alteration with age (10–54 years) were evaluated at the shortest TR_MP2RAGE_ (4000 ms) in white matter (WM), cortical and deep grey matter regions. The T_1_ values were insensitive to the choice of different TR_MP2RAGE_. T_1_ values varied over development into adulthood, especially for the deep grey matter regions whereas only a very small difference was observed for WM T_1_. Effective PD maps were produced which did not show a significant difference between children and adults for the age range included. PD* maps and high precision T_1_ maps were produced at 7T with 0.65 mm resolution in 7:18 min:s from an MP2RAGE protocol, previously optimised for UNI and FLAWS-related contrasts by incorporating the excitation flip angle and inversion efficiency B_1_^+^ effects into the fit. Average WM T_1_ values of adults and children were very similar (1092 vs. 1117 ms) while expected reductions in T_1_ with age were found in deep GM.

## 1 Introduction

Quantitative MRI has an important role in the measurement of tissue microstructure and its alteration as part of healthy development and ageing (Deoni, 2010; Eminian et al., 2018; Lutti et al., 2014) and in a wide range of pathology (Adler et al., 2017; McDowell et al., 2018).

Many different approaches exist for longitudinal relaxation constant (T_1_) mapping (A. G. Teixeira et al., 2020; Brix et al., 1990; J. Cho et al., 2024; Christensen et al., 1974; Crawley & Henkelman, 1988; Deichmann & Haase, 1992; Deoni, 2007; Deoni et al., 2003; Frahm et al., 1986, 1987; Graumann et al., 1986; Gupta, 1977; Hahn, 1950; Helms et al., 2008; Henderson et al., 1999; Homer & Beevers, 1985; Leipert & Marquardt, 1976; Look & Locker, 1968, 1970; Lutti et al., 2014; Ma et al., 2023; Manhard et al., 2021; Sanchez Panchuelo et al., 2021; Wang et al., 2022; Wright et al., 2008); however, a fast and widely used approach at 7 Tesla (T) for human brain is the Magnetization Prepared 2 Rapid Gradient Echoes (MP2RAGE) (Marques et al., 2010) sequence. The MP2RAGE sequence (Marques et al., 2010), which is an extension of the Magnetization Prepared RApid Gradient Echo (MPRAGE) sequence (Marques et al., 2010; Mugler & Brookeman, 1990; Van de Moortele et al., 2009), has been used at 7T to produce 3D structural T_1_-weighted brain images and T_1_ maps. This sequence acquires two Gradient Echo (GRE) images (INV1 and INV2) at two different inversion times (TI_1_ and TI_2_) following a non-selective adiabatic inversion pulse where the time between two consecutive inversion pulses is called TR_MP2RAGE_ (Marques et al., 2010). These two images are combined to form the uniform T_1_-weighted image (UNI) which is insensitive to receive field (B_1_^-^) variability, proton density (PD), and T_2_* effects (Marques et al., 2010; Van de Moortele et al., 2009). T_1_ maps are typically produced using the UNI signal from look up tables (LUTs) which assume uniform B_1_^+^ (Marques et al., 2010). Nonetheless, derived images and quantitative maps are still sensitive to transmit field (B_1_^+^) inhomogeneity effects (Marques et al., 2010).

Even though MP2RAGE scan parameters can be optimised to reduce B_1_^+^ effects, some residual B_1_^+^ bias is likely to be present in the T_1_ maps (Marques & Gruetter, 2013; Van de Moortele et al., 2009). To overcome this problem, Marques and Gruetter (2013) employed the MP2RAGE sequence (Marques et al., 2010) and SA2RAGE sequence (Eggenschwiler et al., 2012) to calculate B_1_^+^ values (Marques & Gruetter, 2013). Look up tables could then be used relating the MP2RAGE signal intensity iteratively to the B_1_^+^ value and the T_1_ (Marques & Gruetter, 2013). The B_1_^+^ effect was considered for the excitation pulses and not for the adiabatic inversion pulse (Marques & Gruetter, 2013).

The MP2RAGE sequence can also be optimised to produce GM-dominant FLuid And White Matter Suppression (FLAWS) images (Beaumont et al., 2019, 2021; Martin et al., 2023; Massire et al., 2021; Müller et al., 2022; Tanner et al., 2012; Urushibata et al., 2019), which can be beneficial for deep GM visualisation and potentially improve lesion detection in multiple sclerosis and focal cortical dysplasia (Chen et al., 2018; Martin et al., 2023; Massire et al., 2021; Müller et al., 2022). It would be advantageous to be able to obtain T_1_ maps from these protocols, but T_1_ estimation is challenging due to the low grey matter (GM) signal in the bias-reduced UNI image which is used to calculate T_1_ unless additional bias-reduced FLAWS-related images such as the FLAWS_hc_ are used for this purpose (Beaumont et al., 2021).

A further clinically relevant quantitative value is proton density (PD) which is defined as the number of MR visible hydrogen protons in tissue (Mezer et al., 2016; Tofts, 2003). PD values have been shown to be altered in multiple sclerosis (Engström et al., 2014; Gracien et al., 2017) and brain oedema from different causes (Shah et al., 2008, Blystad et al., 2017). Quantitative PD maps can be obtained from MP2RAGE protocols; however, to the best of our knowledge, this has not been investigated.

A number of studies have proposed to obtain T_1_ maps by adding an additional GRE block to the MP2RAGE sequence (Marques et al., 2010) named as MP3RAGE: Hung et al. (2013) determined T_1_ by fitting the inversion efficiency (*eff*) of the pulse as a free parameter if the pulse profile information was not available; Rioux et al. (2014) obtained T_1_ and flip angle (FA) information by using three different combinations of the images acquired at different inversion times using a LUT as in Marques et al. (2010); and Olsson et al. (2022) fitted T_1_ and B_1_^+^ simultaneously by using closed-form approximations of the signal equations for small FAs while assuming an *eff* value of 0.96.

In this study, we aimed to determine if high-quality T_1_ and effective PD (PD*) maps could be generated from a 7T protocol with shorter than conventional repetition times (TR_MP2RAGE_), previously optimised to generate UNI and FLAWS-related contrasts in a single scan (Dokumacı et al., 2023). The term effective PD has been used because the T_2_* effects have not been considered (Cercignani et al., 2018; Weiskopf et al., 2013). To achieve this aim, analytical equations that account for partial Fourier acquisitions were derived and fitting was performed using the analytical equations in combination with B_1_^+^ maps while investigating the effects of B_1_^+^ both on the excitation flip angles and the inversion efficiency (*eff*). Results using protocols with different TR_MP2RAGE_ values were compared. Data from children and adults using the short TR_MPRAGE_ (=4000 ms) were acquired, and changes in T_1_ with age were investigated.

## 2 Methods

### 2.1 Theory

The time between two consecutive inversion pulses in an MP2RAGE sequence, TR_MP2RAGE_, includes two GRE blocks, the delay (TA) following the first inversion pulse until the start of the first GRE block, the delay (TB) between the two GRE blocks, and the remaining time (TC) following the second GRE block until the application of the next inversion pulse (Marques et al., 2010). Following the approach of Marques et al. (2010), the MP2RAGE steady-state signal for the longitudinal magnetization and the modified signal equations accounting for the partial Fourier acquisition for the GRE blocks were derived as Dokumaci et al. (2022):

$$m_{z,ss}=\frac{M_{0}\left[\left(\left((1-EA){\left(\cos(\alpha_{1})E1\right)}^{n}\;+\;(1-E1)\frac{1-{\left(\cos(\alpha_{1})E1\right)}^{n}}{1-\cos(\alpha_{1})E1}\right)EB+(1-EB)\right){\left(\cos(\alpha_{2})E1\right)}^{n}\;+\;(1-E1)\frac{1-{\left(\cos(\alpha_{2})E1\right)}^{n}}{1-\cos(\alpha_{2})E1}\right]EC+(1-EC)M_{0}\;}{1+eff{\left(\cos(\alpha_{1})\cos(\alpha_{2})\right)}^{n}e^{-\frac{TR_{MP2RAGE}}{T1}}}$$ (1)

$$GRE_{TI1}=\;M_{0}B_{1}^{-}e^{-\frac{TE}{T_{2}^{*}}}\sin(\alpha_{1})\left[\left(\frac{-eff\;m_{z,ss}}{M_{0}}EA+\left(1-EA\right)\right){\left(\cos(\alpha_{1})E1\right)}^{\frac{n}{3}}\;+\;(1-E1)\frac{1-{\left(\cos(\alpha_{1})E1\right)}^{\frac{n}{3}}}{1-\cos(\alpha_{1})E1}\;\right]:=M_{0}k_{1}$$ (2)

$$GRE_{TI2}=M_{0}B_{1}^{-}e^{-TE/T_{2}^{*}}\sin(\alpha_{2})\left[\frac{\frac{m_{z,ss}}{M_{0}}-(1-EC)}{EC{\left(\cos(\alpha_{2})E1\right)}^{\frac{2n}{3}}}-(1-E1)\frac{{\left(\cos(\alpha_{2})E1\right)}^{\frac{-2n}{3}}-1}{1-\cos(\alpha_{2})E1}\right]:=M_{0}k_{2}$$ (3)

In Equation (1), EA = exp(-TA/T_1_), EB = exp(-TB/T_1_), EC = exp(-TC/T_1_), and E1 = exp(-TR_GRE_/T_1_). TR_GRE_ is the time between the small flip angle excitations in the GRE blocks (n in total). The efficiency of the inversion pulse is denoted as *eff* and defined as Marques et al. (2010) $M_{z,inv}=-effM_{z}(0)$ where M_z,inv_ is the inverted longitudinal magnetisation and M_z_(0) is the initial longitudinal magnetisation. Equations (2) and (3) account for the partial Fourier factor of 6/8 in the first phase encoding (partition) direction, acquired in the innermost k-space loop. This results in a shift of the k-space centre from the middle of the n phase encoding blocks to (n/3 + 1) in the sequence implementation.

To fit T_1_ and M_0_ (PD), a nonlinear least-squares solver (*lsqnonlin*) in MATLAB (R2022b, The Mathworks, Natick, Massachusetts) was used to minimise the cost function: _$\underset{\left\{T_{1},PD\right\}}{\text{min}}\;(f_{1}{\left(seq,T_{1},PD,s_{1}\right)}^{2}+f_{2}{\left(seq,T_{1},PD,s_{2}\right)}^{2})\;$_ for f_1_ = s_1_ - PD*k_1_ and f_2_ = s_2_ - PD*k_2_ where s_1_ and s_2_ are the measured GRE_TI1_ and GRE_TI2_ signals, respectively with the theoretical values of PD*k_1_ and PD*k_2_ from Equations (2) and (3). The term *seq* represents the imaging parameters TR_GRE_, TI_1_, TI_2_, TR_MP2RAGE_, n, α_1_, α_2_ whose values are known. The terms k_1_ and k_2_ are not functions of PD but depend on T_1_ and the sequence parameters.

The fitting “options” for the lsqnonlin function were MaxIterations = 100000; MaxFunctionEvaluations = 400000; FunctionTolerance = 1e-20; FiniteDifferenceStepSize = 1e-2; and OptimalityTolerance = 1e-10. The T_1_ search range was 200 ms to 30000 ms while for the unscaled PD 0 to 100000 was used. The initial values for T_1_ and PD were 1500 ms and 20000, respectively.

### 2.2 Different fits for calculating T_1_

To explore the weighting of the B_1_^+^ effect on the excitation flip angles and the inversion efficiency (*eff*), different fits were performed using the nonlinear least-squares algorithm:

**Fit 1** excluded B_1_^+^ information in the excitation flip angles (α_1_ and α_2_) and assumed *eff* = 1 throughout the whole 3D image. **Fit 2** included B_1_^+^ information in the excitation flip angles (α_1_ and α_2_) but still assumed *eff* = 1. Lastly, **Fit 3** included both the B_1_^+^ information in the excitation flip angles and variable *eff*.

For all fits, the phase information was used to determine the polarity of the magnetization due to T_1_ recovery at the first inversion time (TI_1_).

The effect of TR_MP2RAGE_ on the fits was investigated. In addition, T_1_ maps from the vendor which are determined from a LUT without using B_1_^+^ information (Marques et al., 2010) were included for comparison. The function *colorbarpzn* (He, 2024) running in MATLAB was used to create the blue-white-red colormaps and colorbars.

### 2.3 Data acquisition

All data were obtained under institutional ethical approval (REMAS 8700 and 18/LO/1766) and following informed consent. For all scans, a MAGNETOM Terra 7T system (Siemens Healthcare, Erlangen, Germany) using a single transmit (1Tx/32Rx) coil (Nova Medical) was employed.

The summary of scan protocols is given in Table 1. Most of the data were acquired using the protocol optimised in our previous study (Dokumacı et al., 2023) that confers the benefits of short scan time, high resolution, and high-quality FLAWS and UNI contrast options from a single scan. Firstly, data were obtained with different TR_MP2RAGE_ values in a small group of 4 healthy adults (32 ± 2 years, 1f) to assess the invariance of the T_1_ estimate to the TR_MP2RAGE_ (Table 1, Protocols 1-4). Secondly, data from 7 healthy children (12 ± 2 years, 2f) and 2 more adults making it 6 adults in total (35 ± 9 years, 1f) were obtained to determine if expected age-related T_1_ changes could be observed (Table 1, Protocols 5-6). Protocol 7 had a low sensitivity to B_1_^+^ with a longer TR_MP2RAGE_ (Marques et al., 2010) (TR_MP2RAGE_ = 8000 ms).

**Table 1.** Scan protocol summary.

Protocol	Subjects	TR_MP2RAGE_ (ms)	TI_1_/TI_2_ (ms)	α_1_/α_2_ (°)	Resolution (mm^3^)	Partitions	PI factor	Duration (min:s)
**1**	4 adults	4000	650/2220	5/4	0.65 x 0.65 x 0.65	240	3	7:10
**2**	Same as above	4500	650/2220	5/6	0.65 x 0.65 x 0.65	240	3	8:03
**3**	Same as above	5000	650/2220	5/6	0.65 x 0.65 x 0.65	240	3	8:57
**4**	Same as above	5000	650/2220	5/7	0.65 x 0.65 x 0.65	240	3	8:57
**5**	2 adults	4000	650/2280	4/5	0.65 x 0.65 x 0.65	256	3	8:58
**6**	7 children	4000	650/2280	4/5	0.65 x 0.65 x 0.65	256	4	7:18
**7**	1 adult	8000	1000/3300	4/5	1 x 1 x 1	160	3	12:50

TI_1_/TI_2_ is the time between the inversion pulse and the centre of k-space for the 1^st^/2^nd^ GRE block (Marques et al., 2010). Parallel imaging was employed using GRAPPA (Griswold et al., 2002). Protocols 1-4 used a 6/8 partial Fourier factor in both phase encoding directions while Protocols 5-7 applied it only in one direction (slice partial Fourier). Protocol 7 had low sensitivity to B_1_^+^ but a longer TR_MP2RAGE_.

To determine the effect of B_0_ inclusion on the *eff* of the inversion pulse, a B_0_ map (GRE sequence with 3 echoes TE_1_/TE_2_/TE_3_ = 1.02 ms/2.26 ms/4.08 ms and TR = 10 ms) was acquired in one subject. In addition to the B_0_ map, MP2RAGE data using 2 different protocols (Protocols 5 and 7) and 2 different B_1_^+^ maps (sat_tfl and AFI (Yarnykh, 2007)) were obtained where sat_tfl refers to the 2D B_1_^+^ method that uses a slice-selective preconditioning pulse in combination with a turbo FLASH readout (Chung et al., 2010; Fautz et al., 2008). The sat_tfl sequence had TE/TR = 1.73 ms/4000 ms and 4 x 4 x 4 mm^3^ resolution with an acquisition time of 9 s while the AFI had a nominal FA of 60°, TE=15 ms, TI2/TI1 = 5, 4 x 4 x 5 mm^3^ resolution and took 3:16 min:s.

### 2.4 T_1_ and PD* estimation and analysis

Data preprocessing consisted of the following steps performed in MATLAB (R2022b, The Mathworks, Natick, Massachusetts): 1) using the FLAWS_hco_ images, a brain mask was produced to limit the fit to brain and surrounding cerebrospinal fluid, 2) B_1_^+^ map processing, 3) B_1_^-^ bias field calculation for PD* map generation, and 4) determining the inversion efficiency (*eff*) maps which are explained in detail below.

#### 2.4.1 Producing the brain mask

FLAWS_hco_ images were generated using the INV1 and INV2 images (Beaumont et al., 2021; Dokumacı et al., 2023; Tanner et al., 2012) and converted to NIfTI using the *dicm2nii* function (Li et al., 2016). A brain mask was calculated from a segmentation performed on the FLAWS_hco_ image in SPM12 (Penny et al., 2007). Regions were assigned to be within the brain if they had probability of *p* > 0.99 of being either white matter (WM), grey matter (GM), or cerebrospinal fluid (CSF). The resulting binary mask was edited to produce a closed mask with the application of the *imclose* and *imfill* morphological operators in MATLAB.

#### 2.4.2 B_1_^+^ map processing

B_1_^+^ maps were smoothed using the hMRI toolbox (Tabelow et al., 2019), co-registered, and resliced using the FLAWS_hco_ image (Beaumont et al., 2021; Dokumacı et al., 2023; Tanner et al., 2012) as the reference and the B_1_^+^ magnitude images as the source in SPM12 (Penny et al., 2007). The relative B_1_^+^ values for each voxel were multiplied by the nominal flip angles to define the actual flip angles subsequently used in the fitting process.

#### 2.4.3 B_1_^-^ related bias field calculation

Maps of the B_1_^-^ related signal bias in the PD* fits were produced using SPM12 (Penny et al., 2007) using the following parameters in the *Segment* module: extremely light regularisation (0.00001), Bias FWHM of 30 mm cutoff, and 3 different tissue types were chosen because the fits were masked.

#### 2.4.4 Determining the inversion efficiency (eff) maps

The adiabatic inversion pulse was a hyperbolic secant (Baum et al., 1985; Silver et al., 1984) whose *eff* was determined using Bloch simulations for each spatial location for all subjects independently using the RF pulse information exported from the DICOM files in combination with the B_1_^+^ maps and, where available, B_0_ maps.

### 2.5 Data analysis in FreeSurfer

To determine the T_1_ and PD* values for different brain regions, UNI images were segmented in FreeSurfer 7.3 (Fischl, 2012). For this purpose, the UNI images were masked with the same closed masks that were used in the fitting process. Masked images were denoised using the *DenoiseImage* function (Manjón et al., 2010) in Advanced Normalization Tools (ANTs) (Avants et al., 2009). A variant of the N3 Bias Correction algorithm (Sled et al., 1997, 1998), N4 Bias Correction algorithm (*N4BiasFieldCorrection* function) (Tustison et al., 2010) implemented in the ANTs (Avants et al., 2009) package was used with the default parameters (spline spacing b = 180; convergence c = [50 x 50 x 50 x 50, 0]). The masks were part of the inputs for both functions. Images were reoriented to standard orientation using the *fslreorient2std* function in FSL (FMRIB Software Library) (Jenkinson et al., 2012; Smith et al., 2004).

These images were segmented using the *recon-all* function (Collins et al., 1994; Dale et al., 1999; Fischl, 2004; Fischl & Dale, 2000; Fischl, Sereno, & Dale, 1999; Fischl, Sereno, Tootell, et al., 1999; Fischl et al., 2001, 2002; *Non-Uniform Intensity Correction*, n.d.; Sled et al., 1997, 1998) of FreeSurfer (Fischl, 2012) with -all (to perform all including subcortical segmentations) and -cm (to conform to the maximum resolution) options. All segmentations were checked via visual assessment by using the Subcortical Automatic Segmentation (aseg) and Automatic Cortical Parcellation (aparc) files produced by FreeSurfer overlaid on original T_1_-weighted images. If the UNI image segmentation was not successful, the steps above were applied to the FLAWS_hco_ images to be used as the FreeSurfer input. In one subject, parameters used for all other subjects did not result in a good segmentation and therefore were adjusted—a spline spacing of b = 100 and convergence c = [1000, 0] were used to get an improved result.

The intersection of the FreeSurfer and SPM masks were used for WM while adding another constraint to include the values with T_1_ ≤ 2000 ms to avoid contamination from CSF due to imperfect segmentation; for cortex the additional constraint was T_1_ ≤ 2500 ms. For deep GM regions, only the FreeSurfer segmentations were used because of the high constraint in the SPM segmentation (*p* > 0.99) leading to regions of interest including very few voxels (in the extreme cases without any voxels) but, like the cortex, voxels with T_1_ ≤ 2500 ms were included in the masks. Regional T_1_ mean values and standard deviations (SD) were calculated for the group of subjects who had the TR_MP2RAGE_ = 4000 ms protocols to see how the values within different brain regions change by age and to establish values in children at 7T.

## 3 Results

**Fig. 1.**
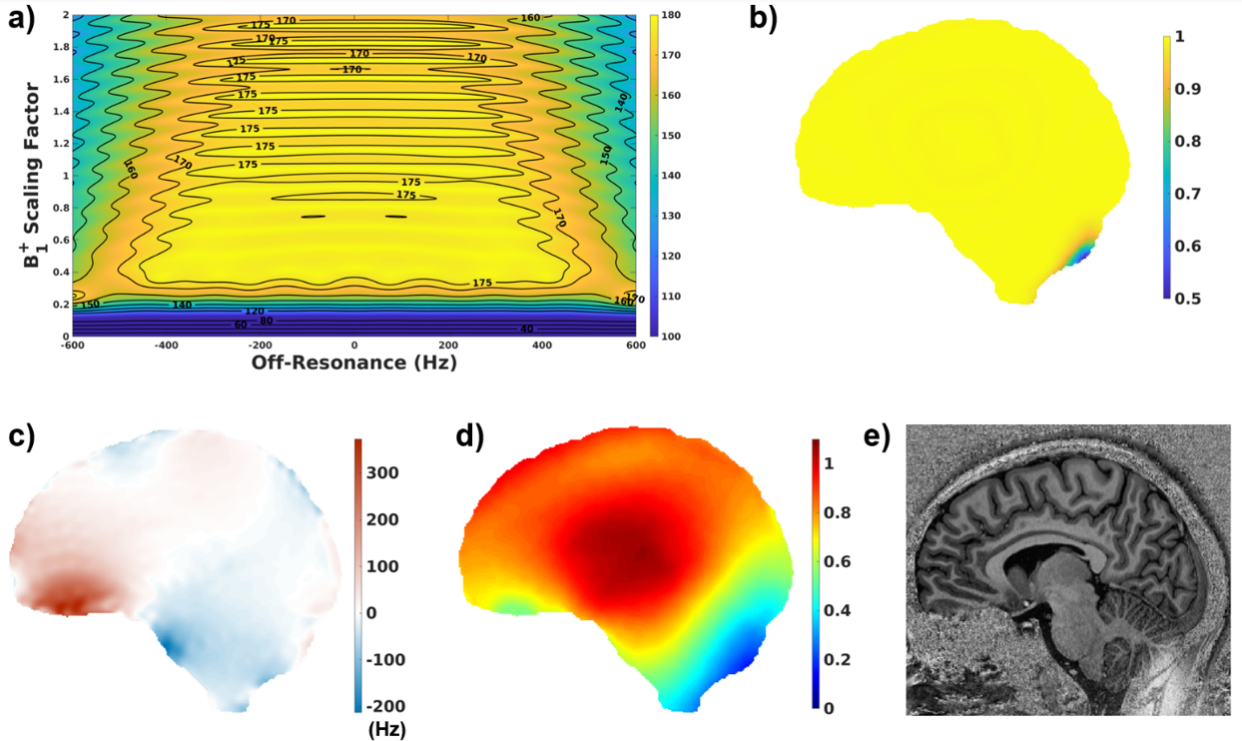
(a) Pulse profile using a range of B_0_ and B_1_^+^ values for this pulse which is insensitive to a large range of B_0_ and B_1_^+^ values (b) Inversion Efficiency (*eff*) map using (c) B_0_ map from a slice acquired in this scan and (d) the corresponding B_1_^+^ map (sat_tfl) (e) the corresponding UNI image which shows a big change at the back of the cerebellum exactly where the *eff* deviates from 1.

The pulse profile with the inversion efficiency simulated using the B_0_ and B_1_^+^ maps from the scan where Protocols 5 and 7 were compared are given in Figure 1. As seen in Figure 1a and b, the inversion pulse is very efficient for a large range of B_1_^+^ and off-resonance values. For this slice, the inversion efficiency mean value ± standard deviation was 0.995 ± 0.023 with a minimum value of 0.477. Without the inclusion of B_0_ information in the inversion efficiency calculations, the inversion efficiency for this slice was simulated as 0.995 ± 0.023 with a minimum of 0.478. Inversion efficiency over the 3D volume was calculated as 0.990 ± 0.048 and 0.990 ± 0.047 with and without the inclusion of the B_0_ map, respectively.

**Fig. 2.**
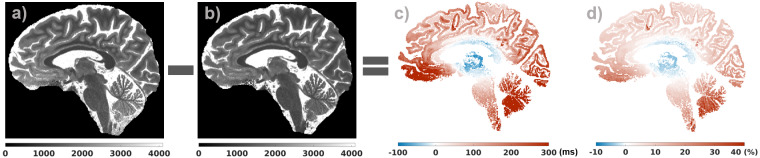
Vendor T_1_ map (a), the fit (b), their difference for WM and GM (c), and the percentage difference with the fit taken as the reference (d) demonstrating the spatial effect related to B_1_^+^ non-uniformity.

The differences between the vendor T_1_ map which is based on a LUT that does not use B_1_^+^ information and the fit that uses B_1_^+^ information demonstrated the expected spatial effect in T_1_ due to the spatial variation in the B_1_^+^ field (Fig. 2). Different fits (Fits 1–3) were performed to investigate the contribution of the *eff* and B_1_^+^ variability to these spatial effects.

**Fig. 3.**
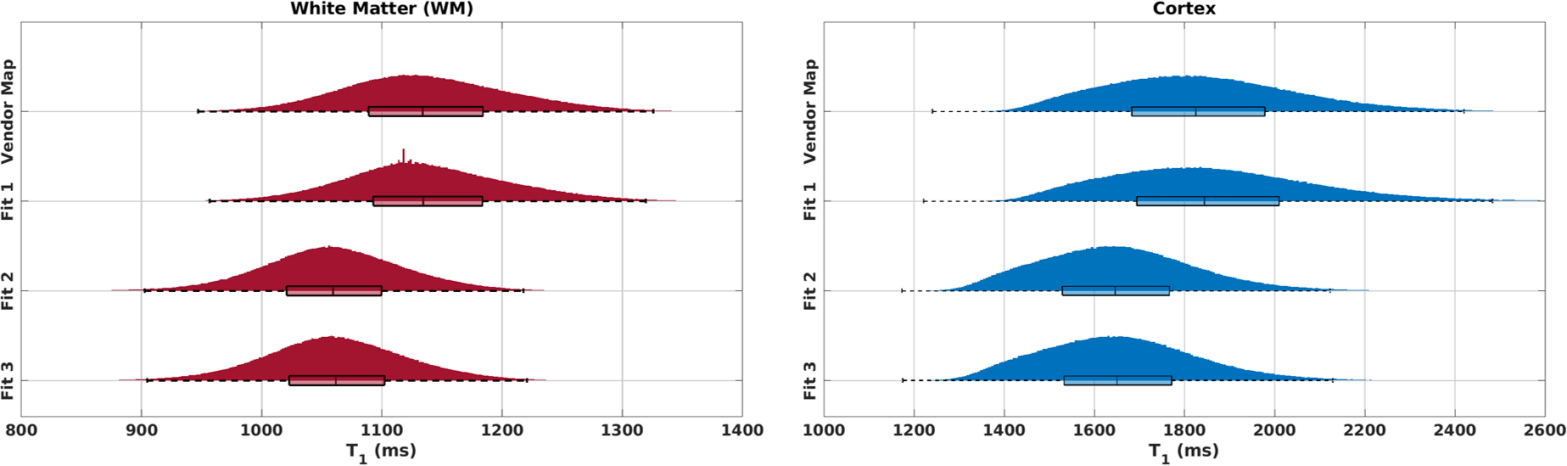
WM and cortex histograms for the vendor T_1_ map and different fits in one adult (Adult 1). Fit 1 does not consider the B_1_^+^ information like the vendor map. Fit 2 considers the B_1_^+^ information but assumes the inversion efficiency to be uniform (*eff* = 1) for each pixel. Fit 3 uses both the B_1_^+^ and *eff* maps for each pixel. Fit 1 produced the closest results to those of the vendor map. The results from Fits 2 and 3 were similar because of the high inversion efficiency of the adiabatic inversion pulse (*eff* = 0.994 ± 0.028 over all subjects).

Figure 3 shows the histograms of WM and cortex from the data of one adult subject using Protocol 1 for these different fits and the vendor T_1_ map. Supplementary Figure S1 shows the histograms from the deep GM regions and Supplementary Figure S2 demonstrates T_1_ maps including the cerebellum using different fits for one slice from the data in Figure 3. Table 2 lists the means and standard deviations for 4 adults including this subject (Adult 1) for WM and cortex.

**Table 2.** Means and SDs for WM and cortex T_1_ values (ms) using different fits for 4 adults (Protocol 1).

T_1_ Value (ms)	Adult 1		Adult 2		Adult 3		Adult 4	
Mean ± SD	WM	Cortex	WM	Cortex	WM	Cortex	WM	Cortex
**Vendor Map**	1138 ± 72	1841 ± 217	1202 ± 81	1957 ± 229	1170 ± 67	1865 ± 227	1187 ± 77	1916 ± 218
**Fit 1**	1139 ± 71	1866 ± 238	1201 ± 82	2021 ± 287	1168 ± 66	1901 ± 267	1184 ± 77	1968 ± 270
**Fit 2**	1060 ± 62	1656 ± 178	1108 ± 67	1723 ± 184	1098 ± 54	1665 ± 191	1103 ± 60	1696 ± 177
**Fit 3**	1063 ± 63	1660 ± 179	1111 ± 67	1729 ± 183	1100 ± 55	1670 ± 192	1105 ± 60	1702 ± 178

As in Figure 3, the vendor map results are closest to those obtained with Fit 1 for all subjects. Likewise, Fits 2 and 3 provided very similar results due to the high inversion efficiency of the adiabatic pulse.

In all subjects, the mean and median values of the vendor map and Fit 1 were the closest as both ignored the B_1_^+^ information. For this highly efficient hyperbolic secant adiabatic inversion pulse with an *eff* of 0.994 ± 0.028 over 13 subjects, the results from Fit 2 and Fit 3 were very similar. Supplementary Table S1 shows the T_1_ results using different fits in deep GM regions where a similar trend is observed.

**Fig. 4.**
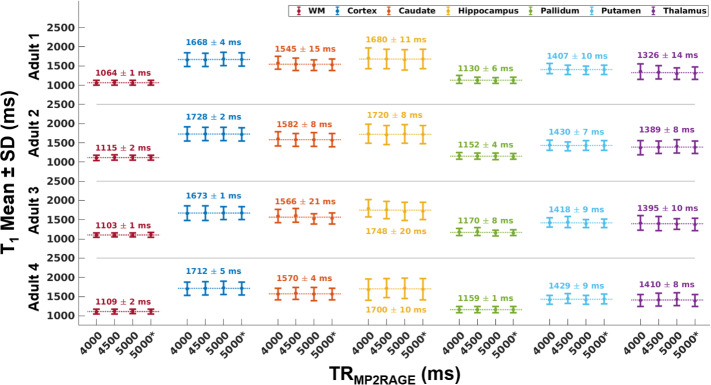
T_1_ measurements using Fit 3 and protocols with different TR_MP2RAGE_ values in 4 adults. The horizontal dotted lines correspond to the mean of the 4 measurements and the mean and uncertainty of the average is given above the line which indicates the small effect of these TR_MP2RAGE_ on the fits. TR_MP2RAGE_ 5000 and TR_MP2RAGE_ 5000* correspond to the protocols 3 and 4 in Table 1, respectively.

To investigate the effect of TR_MP2RAGE_ on T_1_ fits, 4 different TR_MP2RAGE_ protocols (Table 1, Protocols 1-4) were compared using the Fit 3 estimation (accounting for B_1_^+^ effects on both the excitation flip angles and the *eff*). In Figure 4, the T_1_ means and standard deviations for different brain regions with different TR_MP2RAGE_ values are shown. The standard deviation at each TR_MP2RAGE_ corresponds to the physiological variation of the T_1_ over the whole brain. The horizontal dotted lines correspond to the mean across TR_MP2RAGE_. The mean values and the uncertainty of the average using these different protocols are given above the lines. The uncertainty of the average is very small for each brain region in all adults which indicates the high precision of the T_1_ fits across TR_MP2RAGE_.

**Fig. 5.**
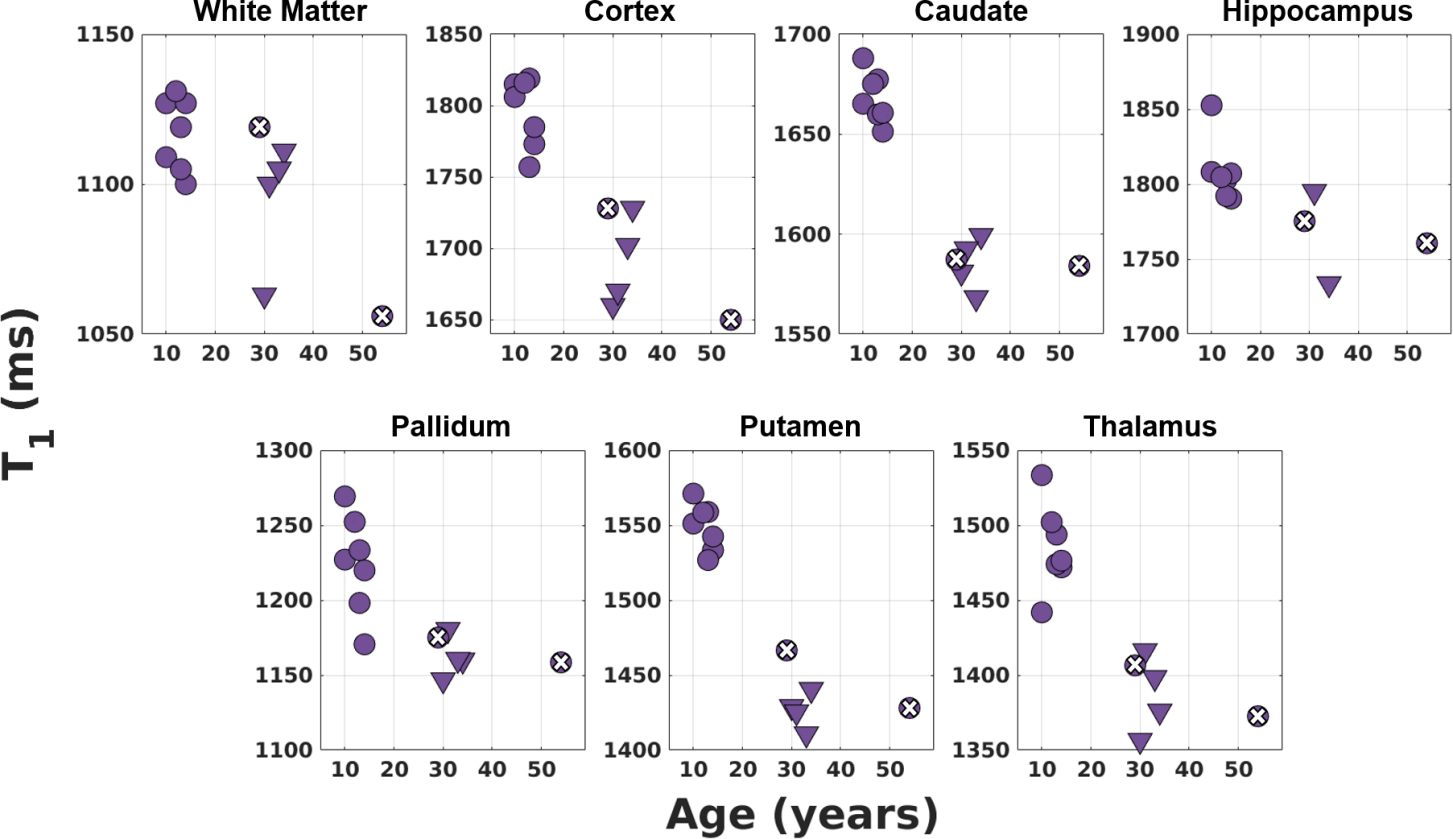
T_1_ fits (Fit 3) using different protocols (triangles correspond to Protocol 1, disks with/without a cross inside correspond to Protocols 5/6—only the PI factor is different—in Table 1) with the same TR_MP2RAGE_ value (4000 ms) in 7 children and 6 adults for different brain regions. The T_1_ values of the children are generally higher compared to those of the adults for the GM regions especially for caudate and putamen. WM values of children (12 ± 2 years) and adults (35 ± 9 years) are very similar.

After investigating the effect of TR_MP2RAGE_ on the fits, data acquired at TR_MP2RAGE_ = 4000 ms were compared in children and adults. Figure 5 shows the results from different protocols (see Table 1 for protocol summaries) for WM, cortex, and deep GM structures in children and adults. The difference between children and adult T_1_s was significant for GM regions especially for caudate and putamen. WM values were very similar for both populations.

**Fig. 6.**
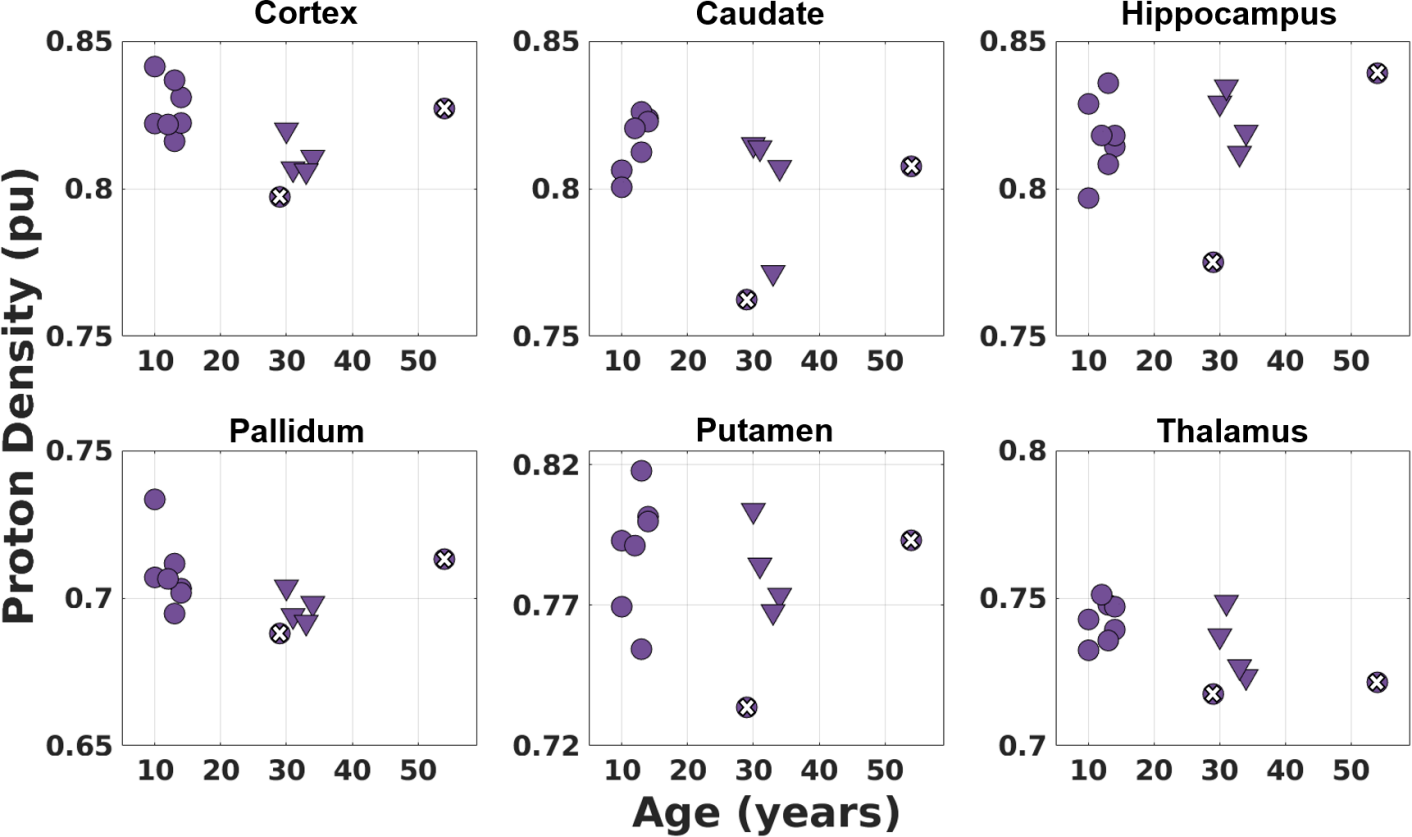
PD* values (Fit 3) using different protocols (triangles correspond to Protocol 1, disks with/without a cross inside correspond to Protocols 5/6—only the PI factor is different—in Table 1) with the same TR_MP2RAGE_ value (4000 ms) in 7 children and 6 adults for different brain regions. There is not a large difference between the children and adult PD* values, although thalamus PD* values suggest a very slight decrease by age. The PD* values were scaled with respect to the WM mean which was assumed to be 0.69 (Weiskopf et al., 2013). No T_2_* correction was made which would include a similar multiplication factor for WM and GM.

The plots for PD* values in different brain regions using different protocols in children and adults are shown in Figure 6. There is not a prominent difference between children and adult PD* values. Table 3 summarises the means and standard deviations for T_1_ and PD* values of the children and adult populations.

T_1_ values were fitted (Fit 3) over the 3D image using 2 different B_1_^+^ maps for Protocol 5 (TR_MP2RAGE_ = 4000 ms) and it was also compared with Protocol 7 (TR_MP2RAGE_ = 8000 ms) which is highly insensitive to B_1_^+^ changes. The B_1_^+^ sensitivities of different protocols used in this study are given in Supplementary Figure S3. The B_1_^+^ maps (sat_tfl and AFI) are shown in Supplementary Figure S4 for a slice with the corresponding T_1_ fits using Protocol 5.

**Table 3.** Means and standard deviations (SD) of T_1_, PD*, and B_1_^+^ in different brain regions of children and adults.

	T_1_ Mean ± SD (ms)				PD* Mean ± SD (pu)		|B_1_^+^| Mean ± SD (pu)	
	Children Fit 3 (12 ± 2 yr)	Adults Fit 3 (35 ± 9 yr)	Children Vendor Map	Adults Vendor Map	Children Fit 3	Adults Fit 3	Children	Adults
**White Matter**	1117 ± 74	1092 ± 63	1221 ± 93	1180 ± 77	0.69 ± 0.04	0.69 ± 0.04	75 ± 13	74 ± 14
**Cortex**	1796 ± 193	1690 ± 184	2128 ± 284	1930 ± 239	0.83 ± 0.08	0.81 ± 0.07	71 ± 13	70 ± 14
**Caudate**	1668 ± 183	1585 ± 171	1722 ± 185	1624 ± 167	0.82 ± 0.06	0.80 ± 0.05	94 ± 6	93 ± 5
**Hippocampus**	1808 ± 261	1740 ± 253	2001 ± 322	1921 ± 293	0.82 ± 0.10	0.82 ± 0.10	83 ± 9	77 ± 10
**Pallidum**	1224 ± 96	1164 ± 96	1235 ± 101	1190 ± 105	0.71 ± 0.04	0.70 ± 0.04	97 ± 3	93 ± 3
**Putamen**	1549 ± 143	1433 ± 131	1649 ± 168	1523 ± 151	0.79 ± 0.05	0.78 ± 0.05	88 ± 5	85 ± 4
Thalamus	1485 ± 202	1388 ± 187	1439 ± 172	1368 ± 165	0.74 ± 0.06	0.73 ± 0.05	105 ± 5	101 ± 6

The standard deviations reflect the physiological variation of tissue T_1_ across the brain. The difference between the T_1_ values of children and adults is significant for GM regions while the WM T_1_ values are very close. The PD* results of children and adults are very similar as well.

**Fig. 7.**
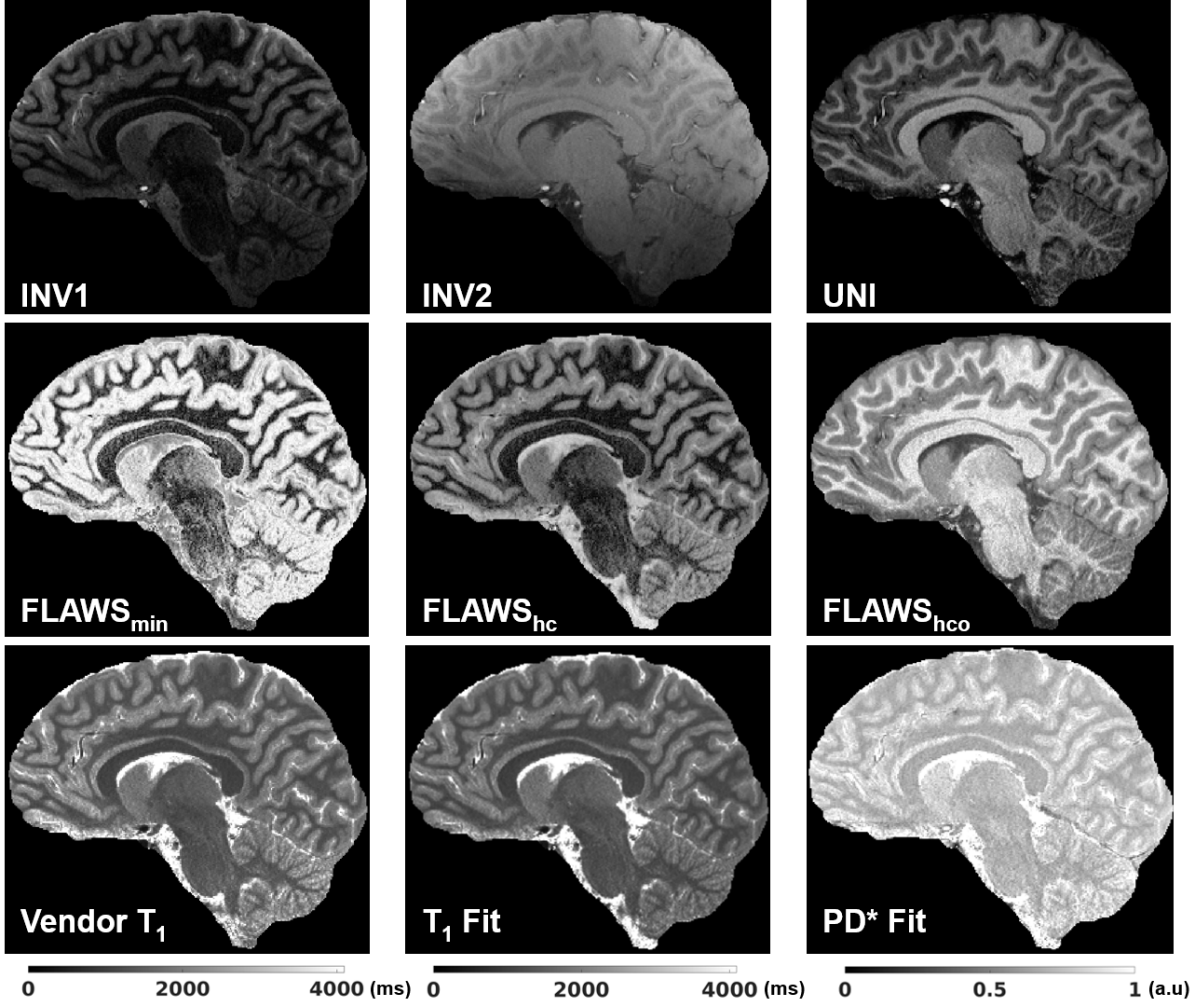
Different contrasts and parameter maps at 0.65 mm isotropic resolution were generated using a single MP2RAGE acquisition in 7:18 (min:s) from a healthy child. The vendor T_1_ map and the T_1_ fit are displayed in the same T_1_ range (0 to 4095 ms). The PD* image has not been corrected for T_2_* effects which would be a small factor due to the short TE (around 3 ms) and similar for WM and GM.

Figure 7 demonstrates representative images and parameters maps that were produced using a single acquisition in under 7.5 min from a healthy child. The vendor T_1_ map and the T_1_ fit are displayed in the same range (0 to 4095 ms). The PD* map was scaled such that the WM mean would be equal to 0.69. No T_2_^*^ correction was made for the PD* images. The T_2_* effect is expected to be similar for WM and GM.

## 4 Discussion and Conclusion

In this study, we improved the precision of T_1_ maps and derived PD* images produced from our multi-contrast protocol that is optimised for UNI and FLAWS-related contrasts in a single scan (Dokumacı et al., 2023) at 7T by including B_1_^+^ effects for both the excitation flip angles and the inversion pulse in the fitting. This protocol made it possible to acquire high-resolution images (0.65 mm iso) in children and adults thanks to its short scan duration (7:18 min:s). Nevertheless, PD* values may still be affected by the method chosen for bias field correction. T_1_ fitting results were consistent at different TR_MP2RAGE_ values (4000 ms, 4500 ms, and 5000 ms) for different brain regions (see Fig. 4). The fits resulted in a mean adult WM T_1_ value of 1092 ± 63 ms which was slightly lower than the values found in literature with the mean range of 1100–1400 ms (Marques et al., 2010; Marques & Norris, 2018; Wright et al., 2008). Mean T_1_ WM value for children (12 ± 2 years) was 1117 ± 74 ms. This result being very similar to the adult T_1_ is not surprising as the myelination is relatively mature after 2–3 years of age with much smaller amplitude development into adulthood (Barkovich, 2000). Cortex and deep GM regions generally had slightly lower values compared to the literature adult T_1_ values (Caan et al., 2019), although they were similar and demonstrated the same pattern of lower T_1_ values in deep GM regions such as the thalamus. It was observed that the GM T_1_ values of children were higher than those of adults especially in deep GM regions such as caudate and putamen. This was consistent with the expected reductions in T_1_ with age from literature (S. Cho et al., 1997; Gracien et al., 2017). Cho and colleagues (S. Cho et al., 1997) demonstrated at 1.5T that T_1_ versus age relationship follows a quadratic curve with minimum T_1_ values observed for deep GM between ages of 38–48 while the inflection in the curve does not happen before 60 years for cortex. Figure 5 shows similar trends for deep GM and cortex with a higher T_1_ in the younger subjects. No significant difference was observed between the mean PD* values of children and adults (Fig. 6; Table 3). This is similar to the results from previous studies for this age range (Hagiwara et al., 2021; Saito et al., 2012). The PD* values in the thalamus (Fig. 6) hint to a very small decrease with age, but a larger study is required to confirm this result. Furthermore, it is possible that these small changes are related to small residual T_2_*-related changes in the PD* measure, despite the relatively short TE. A study by Callaghan and colleagues (Callaghan et al., 2014) found negative correlations between effective proton density and age in the putamen, pallidum, caudate nucleus, and the red nucleus with an echo time of 8.45 ms at 3T.

It was confirmed that the B_1_^+^ has a large effect on the T_1_ values as found by Haast et al. (2021) which necessitates future inclusion of the B_1_^+^ in T_1_ fits. This is particularly relevant where the protocol is sensitive to B_1_^+^ variations at shorter TR_MP2RAGE_ values which are a consequence of the requirements of short scan durations and high resolution. The comparison of the B_1_^+^ corrected high-resolution images (0.65 mm isotropic) to lower-resolution images (1 mm isotropic) acquired with a low-B_1_^+^-sensitive protocol by Marques and Gruetter (Marques & Gruetter, 2013) revealed that by using any suitable B_1_^+^ map, the residual effects of B_1_^+^ variability on T_1_ maps can be removed. The accuracy of the B_1_^+^ map is important as it might affect the T_1_ results significantly for protocols that are more sensitive to B_1_^+^ variations. T_1_ values fitted using two different B_1_^+^ mapping methods in one subject were comparable (Table 4; Supplementary Fig. S4). It could be beneficial to use protocols that are less sensitive to B_1_^+^ variations while satisfying the short scan duration criterion; for instance, by combining CS methods (Candes & Wakin, 2008; Donoho, 2006; Lustig et al., 2007; Mussard et al., 2020; Puy et al., 2012; Trotier et al., 2019, 2022; Vasanawala et al., 2010) with longer TR_MP2RAGE_ values.

**Table 4.** T_1_ value comparison (Fit 3) in the same subject using different B_1_^+^ maps (sat_tfl and AFI) and different protocols.

Brain Region	Protocol	B_1_^+^ Map	|B_1_^+^| (%)	T_1_ Fit (ms)	B_1_^+^ Map Effect (ms)	B_1_^+^ Map Effect (%)	Protocol Effect (ms)	Protocol Effect (%)
**White Matter**	5	sat_tfl	73 ± 15	1119 ± 65	-6	-0.5	-65	-5.8
	5	AFI	74 ± 17	1125 ± 70				
	7	sat_tfl	75 ± 14	1184 ± 59				
**Cortex**	5	sat_tfl	68 ± 16	1728 ± 184	-14	-0.8	-24	-1.4
	5	AFI	69 ± 18	1742 ± 188				
	7	sat_tfl	68 ± 16	1752 ± 121				
**Caudate**	5	sat_tfl	94 ± 5	1587 ± 178	-35	-2.2	-1	-0.1
	5	AFI	98 ± 6	1622 ± 184				
	7	sat_tfl	95 ± 5	1588 ± 143				
**Hippocampus**	5	sat_tfl	71 ± 10	1775 ± 249	26	1.5	-54	-3
	5	AFI	67 ± 12	1749 ± 252				
	7	sat_tfl	71 ± 10	1829 ± 196				
**Pallidum**	5	sat_tfl	92 ± 4	1175 ± 103	-11	-0.9	-55	-4.7
	5	AFI	94 ± 7	1186 ± 106				
	7	sat_tfl	92 ± 4	1230 ± 73				
**Putamen**	5	sat_tfl	84 ± 4	1467 ± 140	-4	-0.3	-33	-2.2
	5	AFI	85 ± 7	1471 ± 144				
	7	sat_tfl	85 ± 4	1500 ± 107				
**Thalamus**	5	sat_tfl	99 ± 7	1407 ± 191	-32	-2.3	-22	-1.6
	5	AFI	102 ± 10	1439 ± 214				
	7	sat_tfl	98 ± 7	1429 ± 159				

Protocol 7 (TR_MP2RAGE_ = 8000 ms) is less sensitive to the variations in B_1_^+^.

Quantitative results are also affected by the accuracy of the segmentations. We used an additional precaution to prevent contamination by CSF voxels by limiting the masks for T_1_ values ≤2000 ms for WM and ≤2500 ms for GM. T_1_ restriction was found to have a negligible effect on all the values except for hippocampus which is an area more prone to segmentation errors (Supplementary Table S2).

One limitation of our study was that the B_0_ map was available for only one subject. However, due to the very high inversion efficiency of this hyperbolic secant adiabatic pulse (*eff* = 0.994 ± 0.028 over all subjects), the simulated B_0_ effect in the inversion efficiency using the map acquired in this subject was negligible. As seen in Figure 1, the efficiency deviates from 1 when B_1_^+^ is very low and it is not so related to B_0_. The B_0_ effect could not be included for the excitation pulses either. However, the effect of B_0_ and B_1_^+^ together on the readout train was simulated (Supplementary Fig. S5) and the T_1_ fit corresponding to Figure 1 was repeated to include the effect of B_0_ on the excitation flip angles. The difference between the T_1_ fits excluding and including this effect is also presented in Supplementary Figure S5. In some local areas such as those close to the sinuses, it affected the results (for instance, by max 8% in the given slice) but the effect was negligible in most of the image.

No separate correction was made for the exponential term $e^{-TE/T_{2}^{*}}$ but TE was very short (about 3 ms). Another limitation was that the signal equations assumed the conventional mono-exponential decay (Labadie et al., 2014; Metere et al., 2017; Rioux et al., 2016). Rioux et al. (2016) showed that longer TI values improve T_1_ accuracy in an MP2RAGE sequence (smaller deviation of the long T_1_ component from the T_1_ value), albeit with increased variance. While the combinations used in our study TI_1_/TI_2_ = 650 ms/2220 ms and 650 ms/2280 ms were outside their simulated range, extrapolating from their results suggests that our parameters would improve T_1_ accuracy without substantially increasing variance. An additional limitation is magnetisation transfer (MT) sensitivity which was not investigated. The original MP2RAGE implementation was calibrated against a single slice long-TR inversion sequence (Marques et al., 2010). However, this validation relied on Agar gel which has very limited MT compared to the human brain (Swanson et al., 2017). Every measurement of T_1_ is sensitive to MT in a different way which makes MT effects very hard to validate in general (A. G. Teixeira et al., 2019).

While multi-slice inversion recovery segmented-EPI or other advanced T_1_ mapping sequences may be more targeted and faster for T_1_ mapping (Sanchez Panchuelo et al., 2021; Wang et al., 2022) and better suited to resolve cortical layers with limited coverage beyond the resolution used in this study, the goal of this study was to obtain the most precise T_1_ and PD* measurements from a sequence that can already provide complementary contrasts such as FLAWS in a single acquisition. In conclusion, it was possible to produce quantitative T_1_ and PD* maps in children and adults at 7T in addition to the UNI and FLAWS-related contrasts from a single scan by using newly derived analytical equations required for partial Fourier acquisitions while incorporating the B_1_^+^ inhomogeneity effect both on the excitation flip angles and the inversion efficiency.

## Data and Code Availability

The datasets that support the findings of this study are available upon request for any reasonable scientific purposes.

## Supplementary Material

Supplementary Material
