# Supplementary material for: Quantitative T1 and Effective Proton Density (PD*) mapping in children and adults at 7T from an MP2RAGE sequence optimised for uniform T1-weighted (UNI) and FLuid And White matter Suppression (FLAWS) contrasts
Source: Imaging Neurosci (Camb). 2025 Nov 13;3:IMAG.a.966. doi: 10.1162/IMAG.a.966 (PMC12616151; doi:10.1162/IMAG.a.966)
Supplement: Supplementary Material [file IMAG.a.966_supp.pdf]

Supplementary Material

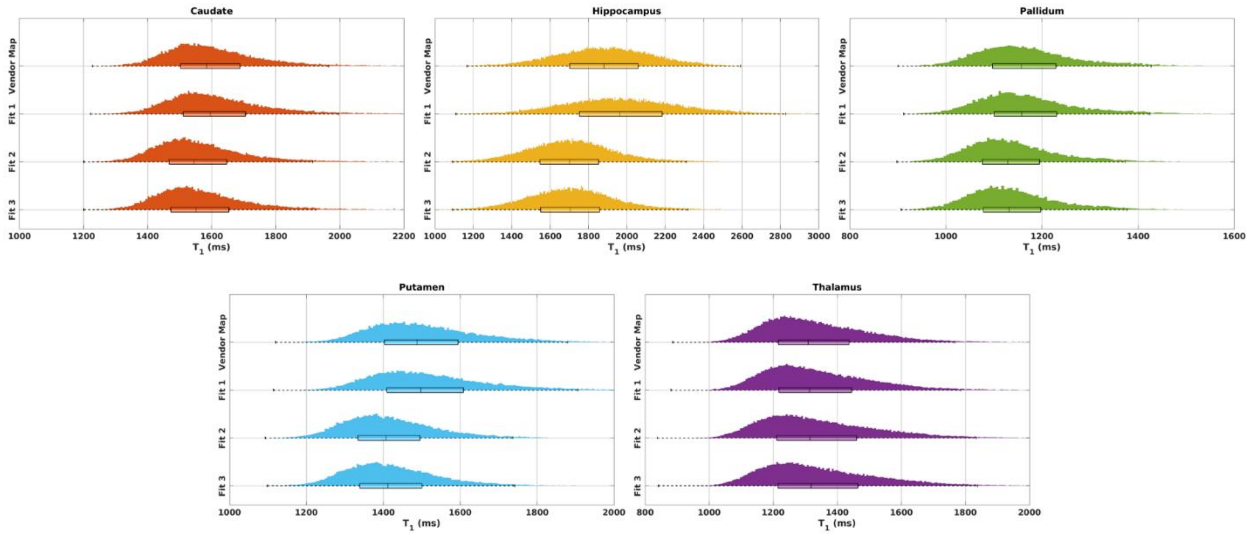

Supplementary Figure S1. T_1 histograms of deep grey matter regions for the vendor T_1 map and different fits in Adult 1. Fit 1 and the vendor map do not consider the B_1^+ information. Fits 2 and 3 both consider the B_1^+ information: Fit 2 assumes the *eff* to be 1 in each pixel whereas Fit 3 uses *eff* information for each pixel.

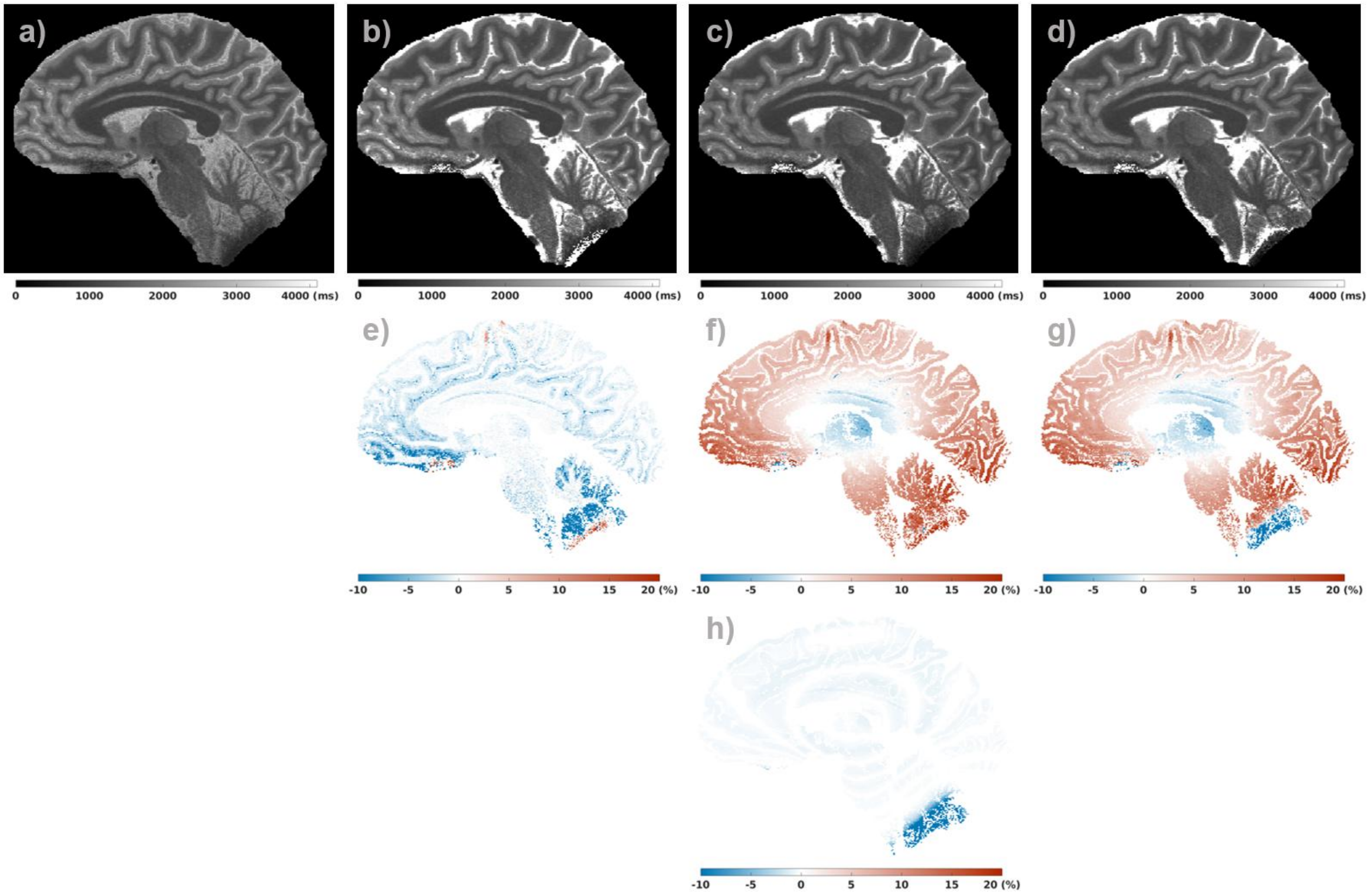

Supplementary Figure S2. T_1 maps and fits for one slice from the dataset in Figure 3: (a) Vendor T_1 map, (b) Fit 1 without B_1^+ correction, (c) Fit 2 using B_1^+ information but assuming *eff* = 1, (d) Fit 3 using B_1^+ and pixel-wise *eff* information, (e–g) percentage differences from (a) for (b–d), respectively, using (a) as the reference, and (h) percentage difference between (c) and (d), referenced to (c).

Supplementary Table S1. Means and SDs for deep grey matter T_1 values (ms) using different fits for 4 adults (Protocol 1) showing the similar trend observed among different fits for WM and cortical T_1 values.

**T_1 Mean ± SD (ms)**					
**Adult 1**	**Caudate**	**Hippocampus**	**Pallidum**	**Putamen**	**Thalamus**
Vendor Map	1610 ± 159	1869 ± 296	1174 ± 114	1511 ± 152	1339 ± 177
Fit 1	1626 ± 173	1966 ± 360	1177 ± 116	1524 ± 165	1347 ± 186
Fit 2	1574 ± 165	1696 ± 265	1144 ± 104	1424 ± 133	1353 ± 200
Fit 3	1581 ± 167	1700 ± 266	1147 ± 104	1429 ± 134	1356 ± 201
**Adult 2**	**Caudate**	**Hippocampus**	**Pallidum**	**Putamen**	**Thalamus**
Vendor Map	1657 ± 182	1902 ± 274	1198 ± 99	1544 ± 153	1364 ± 160
Fit 1	1680 ± 204	1987 ± 342	1200 ± 101	1558 ± 168	1371 ± 199
Fit 2	1594 ± 183	1729 ± 248	1157 ± 90	1437 ± 131	1372 ± 220
Fit 3	1599 ± 184	1734 ± 250	1160 ± 91	1440 ± 131	1376 ± 184
**Adult 3**	**Caudate**	**Hippocampus**	**Pallidum**	**Putamen**	**Thalamus**
Vendor Map	1620 ± 165	1837 ± 233	1183 ± 99	1478 ± 130	1363 ± 154
Fit 1	1631 ± 175	1873 ± 260	1184 ± 99	1482 ± 137	1365 ± 159
Fit 2	1586 ± 171	1788 ± 224	1176 ± 95	1421 ± 120	1409 ± 187
Fit 3	1593 ± 172	1795 ± 225	1180 ± 95	1425 ± 121	1416 ± 191
**Adult 4**	**Caudate**	**Hippocampus**	**Pallidum**	**Putamen**	**Thalamus**
Vendor Map	1574 ± 145	1819 ± 283	1171 ± 80	1465 ± 126	1371 ± 136
Fit 1	1585 ± 152	1840 ± 336	1172 ± 79	1468 ± 131	1374 ± 140
Fit 2	1562 ± 150	1671 ± 274	1157 ± 77	1407 ± 114	1394 ± 153
Fit 3	1568 ± 151	1677 ± 276	1160 ± 78	1411 ± 115	1398 ± 155

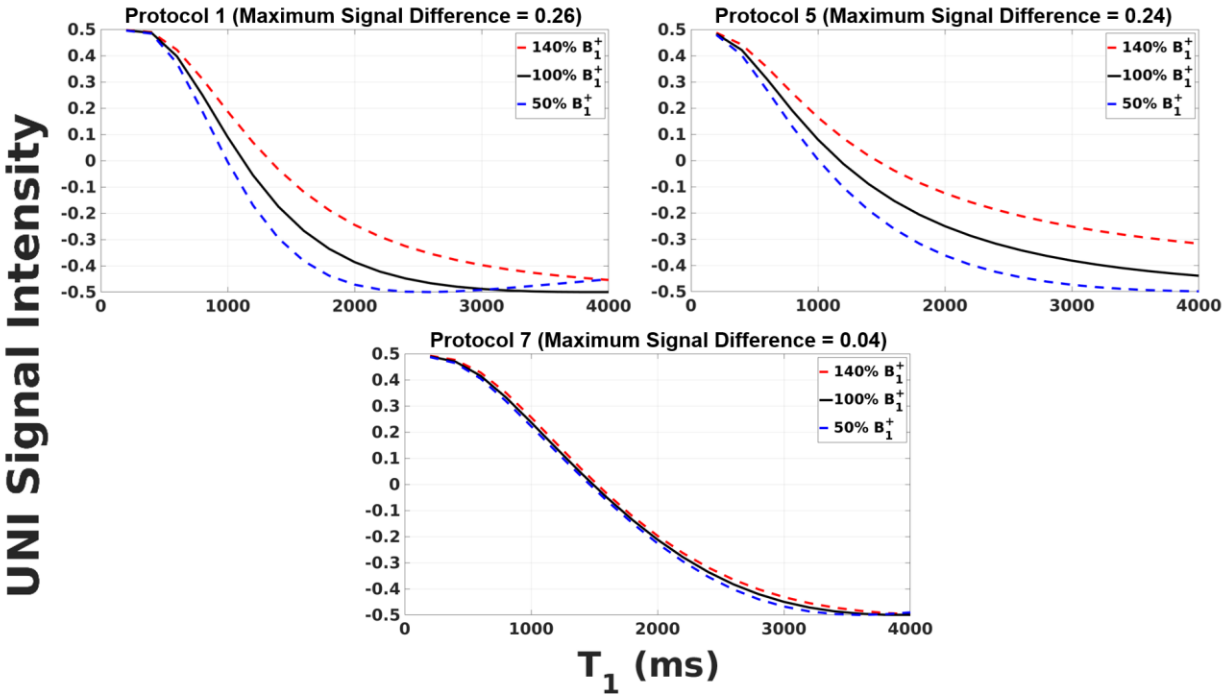

Supplementary Figure S3. B_1^+ sensitivity of different protocols used in this study. It is important to note that Protocol 7 has a $TR_{MP2RAGE}$ value of 8000 ms whereas Protocols 1 and 5 have $TR_{MP2RAGE}$ values of 4000 ms.

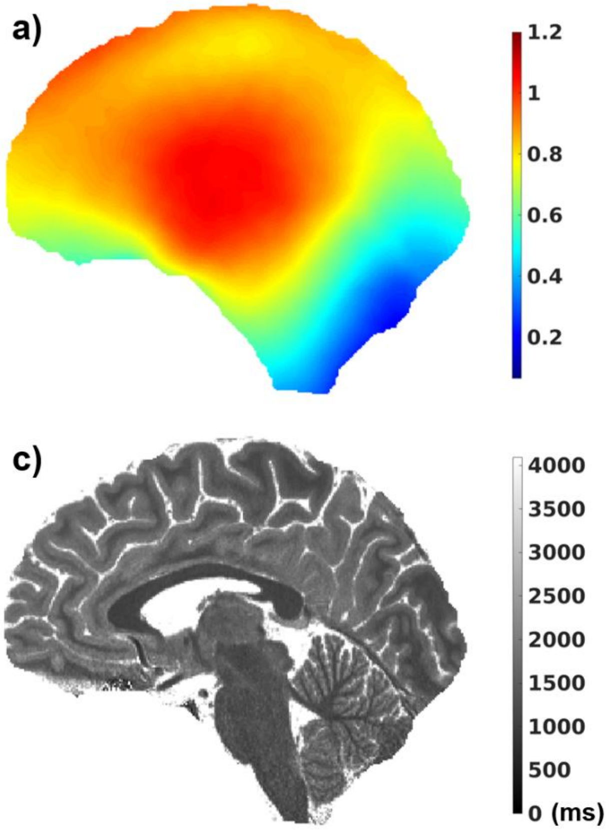

Supplementary Figure S4. Different B_1^+ maps applied in the same subject with the corresponding T_1 fits. a) sat_tfl map b) AFI map c) T_1 fit where sat_tfl was used to correct for B_1^+ d) T_1 fit where AFI was used for B_1^+ correction.

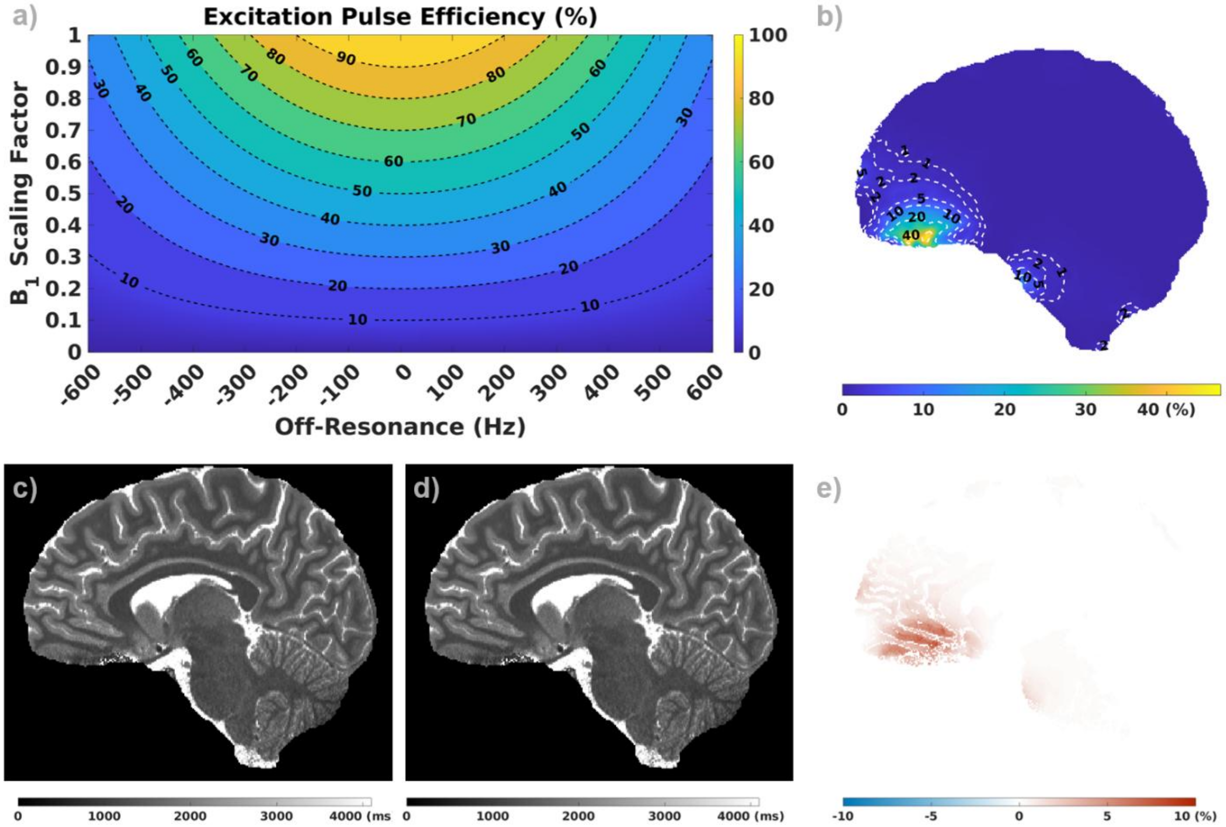

Supplementary Figure S5. a) Excitation flip angles simulated including the effects of B_0 and B_1^+, b) Percentage difference between $\alpha_{excl.B0}$ and $\alpha_{incl.B0}$ using $\alpha_{incl.B0}$ as the reference, c) T_1 fit excluding the effect of B_0 on $\alpha_{exc.}$ and, d) including the B_0 effect on $\alpha_{exc.,}$ e) Percentage difference between c-d using d as the reference.

Supplementary Table S2. Means and SDs of T_1 values in children and adults for different brain regions (ms) using different masks. Intersection Mask refers to the common voxels between the FreeSurfer and SPM (p>0.99) segmentations. The SPM probability value was found to be too limited for deep GM regions sometimes resulting in an empty mask; therefore, only the FreeSurfer mask was used for deep GM. T_1 restriction (≤ 2000 ms for WM and ≤ 2500 ms for GM) was not found to affect the results significantly except for hippocampus for which the CSF contamination was more prominent.

	**Intersection Mask & T_1 Restriction**		**Intersection Mask & No T_1 Restriction**		**FreeSurfer Mask & T_1 Restriction**		**FreeSurfer Mask & No T_1 Restriction**	
	**Children**	**Adults**	**Children**	**Adults**	**Children**	**Adults**	**Children**	**Adults**
**WM**	1117 ± 74	1092 ± 63	1118 ± 126	1093 ± 65	1177 ± 143	1142 ± 124	1185 ± 392	1145 ± 226
**Cortex**	1796 ± 193	1690 ± 184	1805 ± 366	1693 ± 302	1788 ± 225	1698 ± 229	1799 ± 568	1716 ± 551
**Caudate**					1668 ± 183	1585 ± 171	1680 ± 219	1593 ± 201
**Hippocampus**					1808 ± 261	1740 ± 253	1873 ± 560	1786 ± 497
**Pallidum**					1224 ± 96	1164 ± 96	1224 ± 97	1164 ± 102
**Putamen**					1549 ± 143	1433 ± 131	1550 ± 150	1434 ± 138
**Thalamus**					1485 ± 202	1388 ± 187	1491 ± 228	1394 ± 246
